# Barriers to green inhaler prescribing: ethical issues in environmentally sustainable clinical practice

**DOI:** 10.1136/jme-2022-108388

**Published:** 2022-08-18

**Authors:** Joshua Parker

**Affiliations:** Faculty of Health and Medicine, Lancaster Medical School, Lancaster University, Lancaster, UK

**Keywords:** environment, ethics- medical, informed consent, resource allocation

## Abstract

The National Health Service (NHS) was the first healthcare system globally to declare ambitions to become net carbon zero. To achieve this, a shift away from metered-dose inhalers which contain powerful greenhouse gases is necessary. Many patients can use dry powder inhalers which do not contain greenhouse gases and are equally effective at managing respiratory disease. This paper discusses the ethical issues that arise as the NHS attempts to mitigate climate change. Two ethical issues that pose a barrier to moving away from metered-dose inhalers are considered: patients who decline an inhaler with a smaller carbon footprint and increased cost. I argue that while a patient is not morally justified in refusing a more environmentally sustainable inhaler due to the expected harms, a doctor may still prescribe a metered-dose inhaler if they believe that switching without consent might undermine trust or substantially worsen the patient’s health. Turning to cost, I argue that the imperative to combat climate change means the NHS should accept small increased financial costs for lower carbon inhalers, even though this provides no additional direct benefit for the patient. I then go on to consider the implications of the preceding analysis for policy and practice. I argue for a policy that minimises the impact of inhalers on the climate by advocating for a principle of environmental prescribing and explore decision-making in practice. While the arguments here pertain primarily to inhalers, the discussion has broader implications for debates around healthcare’s responsibility to be environmentally sustainable.

## Introduction

In 2020, the National Health Service (NHS) became the first healthcare system in the world to commit to become net carbon neutral by 2040 for emissions under its direct control.^1^ This is now embedded into legislation through the Health and Care Act 2022 and means the NHS has a statutory duty to report on delivering this goal.^2^ This is important because industrialised healthcare is thought to account for 4%–5% of global greenhouse gas (GHG) emissions.^3^ In the UK, the NHS is responsible for 25% of public sector emissions.^4^ The NHS in England produced the equivalent of 25 megatons of carbon dioxide in 2019.^5^ In that same year, atmospheric carbon dioxide was higher than at any time in at least the past 2 million years and this has corresponded with rising global surface temperatures.^6^ Compared with preindustrial levels, we are now living with 1.2°C of global heating.^7^ The Intergovernmental Panel on Climate Change describes the need to limit global temperature change to no more than 1.5°C above preindustrial levels to avoid catastrophe.^7^ To realise this goal in line with the Paris Agreement, scientists estimate that GHG emissions need to halve by 2030 and reach net zero around 2050.^8^ This leaves a remaining carbon budget from 2020 onwards for limiting global warming to 1.5°C with a 50% probability as 500 gigatons of carbon dioxide.^7^ If emission rates are unchanged from today, this carbon budget will likely be exhausted in just less than 7 years.^9^

One important element of the NHS meeting its goal of net zero is mitigating the emissions from metered-dose inhalers (MDIs). These inhalers have a disproportionate carbon footprint compared with other prescriptions because they contain powerful GHGs. This paper analyses the ethical issues raised by the NHS moving away from MDIs to mitigate climate change and its incumbent threats thereby meeting its commitment to net zero.

The paper is divided into three main sections. The first section provides a detailed background on the carbon footprint of inhalers and prescribing practices in the NHS. This lays the groundwork to consider the first question raised by the environmental concerns of MDIs: what objections might be raised to moving away from MDIs because of their carbon footprint? The second section accounts for the bulk of the discussion and responds to this question by identifying patients declining to change inhalers and increased costs as potential barriers to change. First, I consider whether patients are morally justified in choosing to remain on inhalers with a greater carbon footprint. I argue that there is a *pro tanto* duty to switch inhalers and outline two countervailing considerations. Following this, I turn to the issue of increased cost and argue that this should not present a barrier to change. In the third section, I address a second research question: what are the implications of these arguments for policy and practice? Taking the preceding analysis, in the absence of barriers to change, I suggest that NHS targets for the reduction in the environmental impact of inhalers are too modest and the target should be to minimise the environmental impact of inhalers as far as possible. Finally, I reformulate the discussion from the second section of the paper into a principle of environmental prescribing to guide practice and outline a framework to underpin environmentally conscientious clinical decision-making. Here, my focus is on inhaler prescribing; however, this is just one element in a wider discussion occurring across healthcare regarding changes in practice to bring about a net zero NHS. The following arguments use inhalers as a case to help draw out the salient ethical issues, but the discussion can be taken as applying to the broader debate around environmentally sustainable healthcare.

## The carbon footprint of inhaler prescribing

Inhalers form the mainstay of treatment for various respiratory illnesses, primarily asthma and chronic obstructive pulmonary disease. Three types of inhaler are used to manage these: MDIs, dry powder inhalers (DPI) and soft mist inhalers. It is thought that MDIs make up 70% of inhaler prescriptions in the NHS.^10^ MDIs were not always the predominantly prescribed inhaler, however. It has been reported that around 66% of inhaled corticosteroids for asthma were DPIs in 2000 compared with 9% in 2017.^11^ This is higher than in the rest of Europe where MDIs account for approximately 40% of inhaler prescriptions.^12^ In Sweden, for example, MDIs account for only 13% of inhaler prescriptions.^13^ The main reason behind the trend towards relying on MDIs in the UK is cost.^14^

MDIs contain hydrofluorocarbon propellants which are powerful GHGs. For example, HFA-134a, which is found in most MDI inhalers, has a global warming potential of 1300. This means that if we compare the same volume of HFA-134a and carbon dioxide over the same time period, HFA-134a will absorb 1300 times more solar energy than carbon dixoide.^14^ Several different hydrofluorocarbons are used in MDI devices each with differing global warming potential. The primary alternative to MDIs are DPIs which do not require a propellant to deliver the medication. Soft mist inhalers are also propellant free resulting in a lower carbon footprint than MDIs but I will focus on DPIs here even if my arguments still apply to all propellant-free inhalers. To make a simple comparison, it has been estimated that the carbon footprint of a commonly prescribed MDI like salbutamol is similar to driving a mid-sized family car 175 miles (280 km) per inhaler whereas the equivalent DPI inhaler amounts to 4 miles (6 km).^15^ As MDIs are commonly prescribed and contain GHGs, it is easy to see how they alone are responsible for 4% of the carbon footprint of the NHS.^10^

DPIs present a viable alternative to MDIs because they have a substantially smaller carbon footprint and they are similarly effective.^13 14^ Some patients are unable to use a DPI, however. Sufficient negative inspiratory pressure is required to work a DPI, meaning that some children and patients with very severe lung problems will be unable to use one. As many patients can use a DPI and they are no worse for a patient’s health, the NHS has set a target of a 50% reduction in the carbon impact of inhalers by 2028.^16^ Prescribing makes up a significant proportion of the carbon footprint of primary care and inhalers are disproportionately represented.^1^ As most inhaler prescriptions occur in primary care my focus is on general practice.

As MDIs contain GHGs and for many patients do not provide any additional health benefits, moving away from MDIs is an important step in meeting the target of net zero NHS. There are two major strands in achieving this.^i^ The first element concerns short-acting beta agonists. The second is to deprescribe MDIs in favour of DPIs. An important part of the carbon footprint of inhaler prescribing is short-acting beta agonists. These are usually prescribed to relieve acute respiratory symptoms and are especially useful in an exacerbation. It has been reported that two-thirds of patients’ day-to-day asthma treatment is dominated by a short-acting beta agonist MDI.^14^ What this means is that many patients in the UK rely on short-acting beta agonists to manage their day-to-day symptoms rather than using regular preventer therapy to avoid symptoms in the first place and saving a short-acting beta agonist for acute illness. This matters because over-reliance on, and overuse of, short-acting beta agonists is associated with poor asthma control, increased risk of exacerbations and death.^14^ As most prescribed short-acting beta agonists are MDIs, over-reliance results in more than half of the UK’s inhaler carbon footprint being from short-acting beta agonists, roughly three times that of Europe.^17^ In other words, in the UK, patients often rely on therapies that relieve rather than prevent their symptoms resulting in excess use of inhalers that carry a large carbon footprint and put them at risk of complications of asthma. There is therefore a strong case for improving care by establishing patients on effective preventative treatments and reducing the reliance on short-acting beta agonists. This is significant for simultaneously improving health while reducing GHG emissions.

The other thread in reducing the carbon footprint of healthcare caused by MDIs is moving towards DPIs. In patients who can use a DPI there are broadly two circumstances where this might happen. When a patient is being started on an inhaler for the first time the prescriber may opt for a DPI over an MDI. This would occur either if a patient has a new diagnosis or is stepping up treatment for an established diagnosis. Second, patients who are established on MDI inhalers with stable respiratory disease are invited to switch to a DPI. In these two circumstances, because DPIs and MDIs are clinically comparable in terms of effectiveness, this should make no difference to the patient’s health. If, for example, a patient with asthma needs to step up their regular preventor therapy it makes no difference whether the step-up is to an MDI or a DPI insofar as they can use a DPI.

In patients for whom a DPI is appropriate, four scenarios can now be distinguished:

1. A patient is thought to be overly reliant on short-acting beta agonists. Given the risks to the patient, preventor therapy is preferred.
2. A patient has a new diagnosis of a respiratory illness and requires treatment with inhalers.
3. A patient has an established diagnosis of a respiratory illness and requires a step-up in treatment.
4. A patient has an established diagnosis of a respiratory illness and is stable with an MDI. A switch to a DPI might be considered.

In the first three scenarios, the health of the patient should be improved by inhalers, but not necessarily improved more by an MDI over a DPI. The clinical equivalence of the two has meant that historically cost was used as a tie breaker where perhaps now carbon footprint is, or as I will argue should be. The private goods of improved care and the public good of mitigating global warming are so clearly aligned in scenario 1 that pursuing this is a priority and patients and professionals should be supported in this. In the fourth scenario, a direct switch to an equivalent DPI should make no difference to the health of the patient.

Surveys suggest that patients are concerned about the environmental impact of healthcare and are willing to consider changing inhalers based on this.^18 19^ This is promising as when practitioners offer a DPI based on environmental sustainability, patients are likely to be receptive to this. Nonetheless, evidence also exists that patients feel that environmental considerations should not affect treatment decisions.^20^ A common obstacle to a change in practice are when patients decline and there is no reason to think inhalers present an exception. Although many patients are receptive to changing inhalers, some will reject environmental needs as a reason to change. Primary care practitioners might make efforts to explore the patient’s preferences and attempt to persuade them, but some patients will remain steadfast in their desire to use an MDI. Here we can see the first barrier change: a patient is offered a DPI which at the very least makes no difference to their health but carries a higher carbon footprint and they choose an MDI. This next section begins by addressing the moral status of a patient refusal under these circumstances. Following this, the issue of cost is considered as historically this has been the deciding factor in choosing between DPIs and MDIs raising a question over whether increased costs are justified.

## Two barriers to environmentally sustainable prescribing

### Are patients morally justified in refusing?

In modern healthcare, significant value is placed on patient choice because of its connection to autonomy. Standard accounts of autonomy in medical ethics lay out three criteria which must be satisfied to say a patient is autonomous with respect to a decision.^21^ These criteria are: if they act intentionally, with sufficient understanding and free from controlling influence. Where a patient is offered a more environmentally friendly inhaler and they meet the aforementioned criteria with respect to that decision, should the patient decline, then that decision typically ought to be respected out of a concern for autonomy.

One widely accepted exception to autonomous decision-making in contemporary bioethics is, following John Stuart Mill, that this decision will harm others.^22^ The purpose of environmentally sustainable prescribing is to minimise the harms of climate change. If a patient’s refusal results in harm through climate change this presents a moral justification for thinking that a patient should choose an inhaler that does not contain GHGs. This has significant implications for how primary care approaches switching inhalers. If a patient’s refusal is outweighed by harm to others, the task for general practice is: to identify patients on MDI inhalers for whom a DPI is clinically appropriate, switch them, explain why they have been switched and ensure the patient can use a DPI.

The connection between individual GHG emissions and the harms of climate change are complex. This complexity has been summarised in two salient features of climate change: dispersion of cause and effect and fragmentation of agency.^23^ Fragmentation of agency refers to the fact that the actions of a single emitter do not alone cause global warming. Global warming is caused by many individuals and institutions acting in concert over time. Dispersion of cause and effect describes the complex relationship between GHG emissions and the climate system. The effects of global warming can be felt long after emissions were first produced and often in other parts of the world, meaning that cause is both spatially and temporally distributed from effect. Walter Sinnott-Armstrong has taken these features of climate change to undermine any *individual* moral responsibility to mitigate global warming. As an individual’s act of emitting will not alone cause global warming and because of complexity within the climate system through its distributed effects, Sinnott-Armstrong claims individual acts make ‘no difference’ to global warming and so cannot be said to cause harm.^24^ If this is correct and a patient using their inhaler makes no difference to global warming, then it seems difficult to use harm as a reason to dismiss their autonomous refusal.^ii^

One response to Sinnott-Armstrong is that if each instance of a GHG emission makes no difference to global warming, then how does global warming occur?^25^ A peculiar form of causation is required for each additional emission to make no difference and yet the accumulation of emissions to result in climate change. Sinnott-Armstrong cannot be correct that individual emissions make *no* difference, it is more accurate to say that individuals make minimal difference. Still, such a minor causal contribution to global warming that relies on the actions of others might still be viewed as not causing harm. Causal responsibility associated with using an MDI is not sufficient to underpin moral responsibility even if the difference it makes to global warming is more than zero. In response to this kind of argument, Avram Hiller distinguishes actual from expected harm.^26^ Stealing a patient’s inhaler such that their respiratory illness gets worse is an example of actual harm. Expected harm denotes events that increase the chance of somebody being made worse off thereby reflecting the statistical and cumulative nature of global warming. Hiller goes on to argue that individuals ought to minimise expected harm as compared with any other easily available alternative.

Even if MDIs do not directly cause harm, their significant global warming potential increases the risk of climate-mediated harms and so in this way is associated with *expected* harm. The question of how patients and practitioners ought to respond to the emissions of inhalers is important and cannot simply be dismissed as making no difference. Moreover, since there is another easily available alternative (DPIs) then it is *pro tanto* wrong to use an MDI. For some patients, a DPI is not clinically appropriate, so even though MDIs increase expected harm, a DPI is not an easily available alternative for the patient who is unable to use one. In the next section, I discuss morally justified exceptions. For now, as the expected harm of MDIs is greater than DPIs, and because DPIs are an easily available alternative for many patients owing to their comparable effectiveness to MDIs, a patient is *not* necessarily morally justified in declining a DPI. This suggests that patients ought to opt for a DPI where this is an easily available alternative, that is, just as effective at managing their respiratory condition.

### How should doctors respond to patients’ refusals to switch inhalers?

It might be thought that if a patient is not morally justified in using an MDI over a DPI where the latter is clinically appropriate the response is straightforward: the practitioner simply over-rides the refusal and prescribes a DPI. The patient–practitioner relationship is more complex than this, however, and there are further morally relevant considerations.

One important factor is the impact of changing inhalers on trust. Trust is often seen as central to the doctor–patient relationship.^27^ Interpersonal trust can be described as an affective attitude towards another that we can in some sense rely on them.^28^ In healthcare, this translates into a suggestion that the doctor will act in the interests of the patient. Moreover, some suggest that there is an important connection between patient’s consenting and trust. Onora O’Neill argues that ‘Informed consent… is generally important because it can make a distinctive contribution to the restoration of trust’.^27^ This suggests two ways that over-riding a patient’s refusal might threaten trust. The first is that if informed consent is important for trust, then failing to respect a patient’s refusal simply undermines the distinct contribution informed consent makes to trust. The second is that a patient may feel they cannot rely on a doctor who simply disregards their rejection of an offer to switch. They may withdraw trust from the doctor who they see as failing in their fiduciary role to act in their interests as they seem primarily motivated by environmental concerns (or more cynically, NHS targets).

A second related issue is the patient’s interests. It is well established that doctors ought to act in the interests of their patients. There is evidence that suggests switching inhalers without discussion leads to reduced effectiveness and deterioration in asthma control through poor inhaler technique and patient dissatisfaction,^29 30^ not to mention as part of the proceeding issue of trust. If switching inhalers without a patient’s input worsens the patient’s health, there is a failure to promote their interests. Patients’ interests provide a weighty consideration against the *pro tanto* obligation to minimise expected harm. Nevertheless, this does not automatically lead to a patient being able to opt for an MDI because without it they will be worse off. We can distinguish changing treatments without discussion and without consent. The former is the subject of the aforementioned studies^29 30^ and predictably leads to a deterioration in care such that changes without discussion should be avoided. However, there are no studies of the effects of a change in treatment that a patient does not agree with but has been discussed with them. It is possible that non-consensual switches of this kind will result in poor disease control and if this is the case would outweigh the *pro tanto* obligation. Yet, there does appear to be opportunities to mitigate any potential risks through an appropriate safety net, planned follow-up and a proviso that an MDI can be represcribed. There is no expectation that healthcare professionals promote the patient’s interests at all costs, meaning that minimal and considered risks may sometimes be acceptable.

Trust and beneficence are important moral aspects of doctors’ management decisions in addition to environmental concerns. These important considerations represent morally salient reasons to over-ride a duty to choose options that minimise expected harm. As a *pro tanto* obligation, the need to minimise expected harm can, for the doctor, be over-ridden by a need to maintain trust or to protect their patient’s health. Moreover, as is discussed further in the third section, these aspects may be used to shape the available options to minimise expected environmentally mediated harms. Specifying exactly when these conditions will obtain is difficult. Nevertheless, as longer term relationships with patients remain common within primary care, practitioners ought to be capable of gauging where trust is being eroded. In terms of the effects on health, practitioners should weigh the interests of the patient against the expected harms with both parties being prepared to consider changing inhalers because of the expected harms without consent but following a discussion if it is thought that the risk to the patient is manageable and minimal. It may not always be easy to say exactly when the risk is minimal but the interests of the patient are a weighty concern and so any reasonable concern about worsening of the patient’s health will outweigh the *pro tanto* obligation.

### Cost as a barrier to switching inhalers

One reason for hesitation in prescribing a DPI is cost. The issue of whether DPIs are more expensive than MDIs is complex. The most comprehensive study on this question essentially answers that it depends on which inhalers are being swapped.^31^ Changing MDIs to certain brands of DPIs could cost an extra £12.7 million annually for every 10% of MDIs changed. Targeted prescribing of the cheapest equivalent DPI instead of certain MDIs could lead to cost savings: £8.2 million annually for every 10% of MDIs. While some of these costs could cancel each other out depending on the changes, there is certainly the potential for DPIs to increase costs for the NHS. What makes the calculation especially difficult is accounting for wider effects of switching like reducing exacerbations of chronic respiratory illness and hospitalisations, which also carry a cost. The answer to the health economics of maximising DPI prescribing is far from settled. If it turns out that environmentally sustainable inhalers are costly this will pose a substantial barrier to change. I consider whether increased financial costs are justified.

Typically, we might think that the NHS is liable for higher costs where this brings about sufficient health benefits. As DPIs are just as effective as MDIs there is unlikely to be any additional direct health benefits to the patient. Nonetheless, there may be wider health benefits. Climate change mitigation carries health benefits so it might seem justified for the NHS to accept these costs. However, most of these benefits will be felt in low-income and middle-income countries. Any health benefits of climate change mitigation for the UK are likely to occur long after inhalers are changed. As climate change is disproportionately caused by wealthy countries and the harms felt most by the disadvantaged, this has led many to agree that wealthy countries ought to bear the burden of paying the costs of climate change mitigation.^32^ Nevertheless, it is not obvious that healthcare should accept such costs, even if it is a healthcare system in a wealthy country. We might ask whether it is fair for a healthcare system to accept higher costs to reduce its carbon footprint if this makes no direct difference to patients locally, instead producing spatially and temporally distributed benefits. This raises a question of distributive justice regarding the fair share of the benefits and burdens of tackling climate change for a publicly funded healthcare system. Clearly, this is a rather broad question, but it is highly relevant in considering the topic at hand. Space limits an in-depth discussion and hence my comments are necessarily brief and focused on inhalers. Nevertheless, some of the arguments may have broader relevance to discussions of healthcare’s role in mitigating climate change.

The question of how to share the burdens of climate change mitigation is an issue of ‘burden sharing justice’.^33 34^ Broadly, philosophers have discussed two principles of burden sharing justice to assess what a fair share is: a polluter pays principle and ability to pay. Each of these could be used to justify the NHS accepting higher costs for environmentally sustainable inhalers in the absence of a direct health benefit and so I will discuss each in turn.

‘Polluter pays’ is a straightforward principle that states that those causing global warming should pay the costs of fixing the problem. Henry Shue explains the reasoning behind this:

All over the world parents teach their children to clean up their own mess… If whoever makes a mess receives the benefits and does not pay the costs, not only does he have no incentive to avoid making as many messes as he likes, but he is also unfair to whoever does pay the costs.^35^

Unmitigated global heating will be paid for disproportionately by those who are vulnerable and contributed the least. This principle applies easily to the problem at hand: if MDIs contribute to global warming, then the NHS should pay the cost of mitigation including the cost of using more expensive inhalers.

Two issues arise with a polluter pays principle. The first is that it is not entirely obvious that the NHS is the polluter. Perhaps it is, but plausibly it could also be the patient using the MDI, the prescriber, the pharmaceutical company and so forth. If we are not convinced that the NHS is the polluter, it may be considered unfair that the NHS pays. Worse still, it is difficult to make the NHS as an institution pay because the funds for more expensive inhalers must be found either through the taxpayer or from elsewhere within the NHS. As it is patients or taxpayers who ultimately pay, we may be especially concerned by a disconnect between who the polluter is and who pays. This leads to the second concern. Some claim that ‘subsistence’ as opposed to ‘luxury’ emissions are an exception to a polluter pays principle. Shue argues that to treat all emissions as equal regardless of their purpose is to ‘ignore the fact that some sources [of greenhouse gas emissions] are essential and even urgent for the fulfilment of vital needs and other sources are inessential or even frivolous’.^36^ It might seem obvious that GHGs emitted in the pursuit of respiratory health are the very epitome of subsistence emissions and therefore the NHS is not liable for the costs. If health emissions are considered subsistence and therefore exempt, then MDIs are the obvious choice for they are just as effective and perhaps cheaper. The distinction starts to fall apart depending on how we interpret ‘essential’, however. Most would agree that health is a vital need, the problem is whether MDIs are *essential* when DPIs are also widely available and carry a lower carbon cost. MDIs are not essential to fulfil this vital need in the sense that we can achieve the same health ends with DPIs, but MDIs are essential if the costs are especially high in the context of a resource-limited NHS.

The concept of subsistence emissions seems to go too far in idealising any health-related emissions as essential. However, this does clarify two important insensitivities of a polluter pays principle. First is an insensitivity to the vital needs that healthcare fulfils generally. The second is the magnitude of the costs that healthcare might be forced to pay by accepting a polluter pays principle. Taken together, the problem is that simply saying that healthcare ought to pay to fix its own mess might force healthcare into accepting higher costs that ultimately limit its ability to meet its wider morally valuable goal of protecting and promoting health. If changing inhalers is very costly and healthcare pays because of a polluter pays principle, funds spent on climate mitigation cannot be spent on healthcare elsewhere creating the potential for opportunity costs. MDIs are then *essential* in helping a healthcare system meet a vital need with limited resources.

An ability to pay principle can be used to avoid these problems. Rather than polluters paying in proportion to their emissions, or whatever the costs, responsibility could be distributed based on an ability to shoulder the cost. 'Cost' is usually conceived of as financial costs, so the wealthy pay. One might object at this point that ability to pay does not take us any further. It is well known that the NHS is underfunded and struggling to meet demand after years of austerity, and now a pandemic. When one looks to the wealthy to pay the bill of mitigating climate change, the NHS hardly features highly. If ‘ability’ means spare or large amounts of wealth, it seems the NHS does not have the *ability* to pay for more expensive environmentally sustainable inhalers given the current political context.

This interpretation sets the bar too high on ‘ability’. Rather than ability, meaning that one is easily able to pay to mitigate climate change, it could have a more moderate reading. To say one has an ability to pay means that one has sufficient resources to mitigate climate change without having to sacrifice too much of comparable moral importance. In this way, ability to pay has much in common with a ‘duty of easy rescue’.^37^ This states that if the cost of engaging in some activity is small but the harms averted great, then one is obligated to act. A more moderate ability to pay principle suggests that the NHS has a duty to pay the costs of mitigating climate change insofar as the costs are not too significant given the grave nature of unmitigated climate change. In the overall NHS budget, the resources needed to pay for DPI devices are not particularly burdensome based on the calculations from Wilkinson *et al*.^31^ Even if they were higher, the NHS should be prepared to accept these costs given the importance of preventing global warming insofar as this does not significantly threaten the NHS ability to protect and promote health.

## Greener practice

This section explores the implications of the preceding arguments for policymakers and practice. One important conclusion of my arguments is that an NHS target of a 50% reduction in the impact of inhalers might be thought of as too modest. It is undeniable that there is a desperate need to limit GHG emissions and healthcare makes a significant contribution to these globally. As many patients are able to use a DPI and we know that other European countries have a substantially lower volume of MDI prescribing than the NHS, there is clearly scope to reduce MDIs to only those that are clinically indicated. Whilst cost is clearly an important consideration and so policymakers should take account of the costs of switching inhalers, I have argued that higher costs alone do not rule out switching. The NHS should accept higher costs insofar as they do not have to sacrifice too much of comparable moral value to mitigate the GHG emissions from inhalers. The goal for policymakers therefore is to minimise the environmental impact as far as possible.

This leads to a question of how healthcare professionals and patients can help minimise the environmental impact of inhalers in practice. The analysis from sections ‘Are patients morally justified in refusing?’ and ‘How should doctors respond to patients’ refusals to switch inhalers?’ can be summarised into the following principle of environmental prescribing to help guide practice:

It is *pro tanto* wrong to choose a treatment which produces an expected amount of harm greater than any other equally clinically effective alternative unless: (1) this might undermine trust; or, (2) it significantly worsens a patient’s health.

What this principle does is asks doctors and patients to look at the available options and, where these are equally effective, pick the one that minimises expected harm. Those who are not able to use a DPI, for example, those with severe respiratory illness who cannot generate sufficient pressure to inhale the powder, are not obligated to switch because for them an MDI is the most clinically effective. Otherwise, this principle neatly summarises both why patients who can use a DPI are morally obligated to choose this over an MDI as well as both why and when practitioners may nevertheless permissibly prescribe an MDI. Of note, this principle might also apply to other problems in clinical practice related to environmental sustainability like anaesthetic gases or those with a similar structure, like antibiotic prescribing.

The principle of environmental prescribing makes clear that in practice, where an MDI is unnecessary, the default option ought to be a non-propellant containing alternative like a DPI. What this means is that primary care clinicians, when presented with a patient in one of the four scenarios from the first section, should support patients to avoid MDIs. While patients are receptive to change for environmental considerations, changes to the management of a chronic condition can be distressing. How these conversations occur is important in moving towards more environmentally sustainable respiratory care, and sensitive, patient-centred communication will underpin making decisions that are good for the patient and good for the planet.

In some cases, a patient will not be persuaded to try a DPI and the effect on the practitioner–patient relationship or the interests of the patient may mean an MDI is chosen. This is not the end of the matter as there remains more than can be done to minimise the environmental impact of the MDI. The duty implied by the principle of environmental prescribing is to make management choices that minimise expected harm *given the options*. Patients cannot necessarily be forced to trial a DPI and so the options are reshaped by a need to maintain trust and act in the patient’s interests. The next step then is to re-examine the clinically effective options that minimise expected harm. This means making every puff of an MDI count. If a patient insists on using an MDI, to discharge the duty implied by the principle they must: have excellent inhaler adherence, have excellent inhaler technique, not overuse medication, count MDI puffs to ensure they are not overordering inhalers, take empty MDIs to be disposed of in an environmentally sensitive manner and undertake other actions to protect their respiratory health to minimise inhaler requirements. Many of these actions are good for the patient’s health, meaning the patient has prudential reasons to undertake these. Consequently, this poses little risk of overburdening the patient nor does it threaten trust. For the patient with a strong preference for an MDI rather than a green alternative, they have an additional reason to undertake this beyond their own health: it is better environmentally and reduces expected harm. It is important to explain to the patient that a need to protect the environment means that prescribing an MDI generates further obligations for them. This level of commitment may not be possible for every patient who refuses a DPI. It may be the case that minimising the impact of MDIs on the climate while still maintaining health means some of these actions are tailored to the individual patient keeping in mind what is easily available for them. A second example of minimising expected harm while prescribing an MDI regards scenario 1 from the first section. Recall a patient who is overusing short-acting beta agonists. Ideally, the patient would start a DPI preventer; however, any preventer including an MDI will still have a smaller carbon footprint. Starting a preventer therapy is so clearly in the patient’s interests that if an MDI is preferred, what is in the patient’s interests shapes what options are available to minimise expected harm. It should be apparent that there are various ways of minimising expected harm within the confines of maintaining trust and acting in the patient’s interests and the principle of environmental prescribing is useful in guiding this.

## Conclusion

The climate crisis is a ‘code red for humanity’ given it threatens everything we care about.^38^ It has been estimated that for every 10% of MDIs changed to DPIs, 58 kilotons of carbon dioxide equivalent could be saved annually in England.^16^ This makes the task of changing inhalers both urgent and important. In this paper, I address two potential barriers to change: patient refusals and higher costs. I have argued that while both these considerations are important, neither poses an insurmountable barrier to change. Considering the number of patients who could use a DPI, there is a substantial opportunity to reduce the environmental impact of inhalers. In the latter part of the paper, I discussed what the arguments presented here might mean for policy and practice. While the discussion here has primarily focused on inhalers, this serves as a case to motivate discussion of the underlying ethical issues in the relationship between healthcare and environmental sustainability and hence the arguments here serve a greater purpose in considering the responsibilities of healthcare in responding to the climate crisis.

## Data Availability

No data are available.

## Footnotes

There is a third option which involves developing alternatives which contain propellants with lower global warming potentials. This is an important option because there are patients who are not able to use a DPI and so having an MDI with a lower carbon footprint would be useful. This is not discussed further here because it will be years before they are prescribed in practice. See Wilkinson and Woodcock^14^ for more.

It is worth noting that the objection that inhalers make ‘no difference’ to global warming could be raised as an independent objection to the project of green inhaler prescribing. One might argue that inhalers should not be changed based on environmental considerations at all because on a small scale it makes no difference. Of course, the NHS is changing inhalers at scale and is likely to, therefore, make a difference. Moreover, making no difference alone does not provide a particularly strong reason not to change, if it truly makes no difference why complain either way? The argument from it not making a difference is strongest in connection to some other morally important concern like a patient refusing and hence is explored in connection to refusals.
